# Anti-nociceptive, anti-inflammatory and toxicological evaluation of Fang-Ji-Huang-Qi-Tang in rodents

**DOI:** 10.1186/s12906-015-0527-5

**Published:** 2015-02-05

**Authors:** Yu-Chin Lin, Ching-Wen Chang, Chi-Rei Wu

**Affiliations:** Department of Biotechnology, TransWorld University, No.1221, Zhennan Rd., Yunlin County, Douliu City, 64063 Taiwan; Department of Cosmetic Applications and Management, Mackay Junior College of Medicine, Nursing, and Management, No. 92, Shengjing Road, Beitou District, Taipei 11260 Taiwan; Department of Chinese Pharmaceutical Sciences and Chinese Medicine Resources, College of Pharmacy, China Medical University, No. 91, Hsieh Shih Road, Taichung, 40402 Taiwan

**Keywords:** Fang-Ji-Huang-Qi-Tang, Radix Stephania Tetrandra, Tetrandrine, Antinociceptive, Anti-inflammatory, Subacute toxicology

## Abstract

**Background:**

Fang-Ji-Huang-Qi-Tang (abbreviated as FJHQT), composed by six medicinal herbs including Radix Stephania Tetrandra, Radix Astragali, Rhizoma Atractylodis Macrocephalae, Radix Glycyrrhizae, Rhizoma Zingiberis and Fructus Ziziphi Jujubae, is a frequently Chinese prescription for treating painful and inflammatory disorders such as rheumatoid arthritis. When Radix Stephania Tetrandra was misused with Aristolochia species, acute or chronic nephropathy caused by aristolochic acid was happened. Thus, the present study was aimed to identify Radix Stephania Tetrandra and performed the pharmacological and toxicological evaluation of FJHQT extract in rodents.

**Methods:**

Radix Stephania Tetrandra was identified by macroscopic and microscopic observation, and the content of tetrandrine in FJHQT extract was measured by high performance liquid chromatography. Then, the pharmacological activities of FJHQT extract with respect to clinical use was investigated with acetic acid-induced writhing response, formalin-induced licking response and carrageenan-induced paw edema. Finally, we evaluated the subacute toxicology of FJHQT extract after 28-day repeated oral administration in rats.

**Results:**

Radix Stephania Tetrandra was correctly used in FJHQT extract, and the content of tetrandrine in FJHQT extract was 2.5 mg/g. FJHQT extract produced a pronounced and dose-dependent antinociceptive and anti-inflammatory effects in three above models. FJHQT extract after 28-day repeated administration did not caused any hematological, biochemical and histological change in rats.

**Conclusions:**

We suggest that FJHQT extract is a high safety index Chinese medicine for antinociceptive and anti-inflammatory application when Radix Stephania Tetrandra was correctly used in FJHQT. Its antinociceptive and anti-inflammatory mechanism might be related to peripheral nociceptive pathway such as prostaglandins.

**Electronic supplementary material:**

The online version of this article (doi:10.1186/s12906-015-0527-5) contains supplementary material, which is available to authorized users.

## Background

Fang-Ji-Huang-Qi-Tang (abbreviated as FJHQT), a frequently Chinese remedy for relieving pain and edema from abdominal pain and rheumatoid arthritis, is composed of Radix Stephania Tetrandra (the roots of *Stephania tetrandra*), Radix Astragali (the roots of *Astragalus membranaceus*), Rhizoma Atractylodis Macrocephalae (the roots of *Atractylodes macrocephala*), Radix Glycyrrhizae (the roots of *Glycyrrhiza uralensis*), Rhizoma Zingiberis (the roots of *Zingiber officinale*) and Fructus Ziziphi Jujubae (the fructus of *Ziziphus jujuba*) [1]. Pharmacological reports indicated that FJHQT possessed hepatoprotective, renal-protective, and immune-modulatory activities [1]. Many reports pointed out that Radix Stephaniae Tetrandrae and Radix Astragali possessed anti-inflammatory activities *in vitro* and *in vivo* [2-6]. However, no scientific report regarding the antinociceptive and anti-inflammatory activities of FJHQT has been published. Therefore, the present study was attempted to investigate the antinociceptive and anti-inflammatory effects of FJHQT extract in rodents with acetic acid-induced writhing test [7], formalin-induced licking test [8] and carrageenan-induced edema test [9].

The dry roots of Aristolochia plants are being used as an alternative for Radix Stephaniae Tetrandrae in China and European for a long time, but the former contains aristolochic acid to cause acute and chronic nephropathy. Therefore, the present study was measured the content of tetrandrine in FJHQT extract by high performance liquid chromatography (HPLC) and evaluated the subacute toxicology of FJHQT extract after 28-day repeated oral administration.

## Methods

### Preparation of plant extract

Radix Stephania Tetrandra (TWU-Plantec-FIJHQT-0001), Radix Astragali (TWU-Plantec-FIJHQT-0002), Rhizoma Atractylodis Macrocephalae (TWU-Plantec-FIJHQT-0003), Radix Glycyrrhizae (TWU-Plantec-FIJHQT-0004), Rhizoma Zingiberis (TWU-Plantec-FIJHQT-0005) and Fructus Ziziphi Jujubae (TWU-Plantec-FIJHQT-0006) were purchased from Taiwan market and identified with macroscopic and microscopic method by Professor Lin Y. C.*.* These characteristics include vascular bundles and the morphology and distribution of vascular bundles, catheters and fibers *etc.*. These above medicinal materials were deposited in the Department of Biotechnology, TransWorld University. FJHQT (17.5 kg) was composed of these above medicinal materials at a ratio of 10:10:6:3:3:3. They were washed separately and put in the oven at 70°C to dry until its water content is below 5%. Then they were cut into thin slices and put into a container (100 × 100 × 100 cm^3^). Distilled water was added and heated at 100°C for 60 minutes consecutively. The solution was dried and concentrated with a rotary evaporator (Laborota 20 compact), purchased from Heidolph Instruments GmbH & Co. (Schwabach, Germany), under 50°C and 120–180 mbar. The extract was grounded into powder, and sealed into a Pyrex glass bottle. The yield is about 32.8%.

FJHQT extract (25, 50, 100 mg/kg) were dissolved in sterile distilled water and administered orally 60 minutes prior to the injection of an inducer. Control were received sterile distilled water in the same experiments. Indomethacin (INDO) (10 mg/kg) were prepared as suspension with 0.5% carboxymethylcellulose and administered intraperitoneally 30 minutes prior to the injection of an inducer.

### Subjects

Male Sprague–Dawley rats (200–250 g) were used for the study of anti-inflammatory activities and toxicological evaluation. Male ICR mice (20-25 g) were used for testing the analgesic effects. The experimental protocol (Protocol No. 98-113-NH) was approved by the Institutional Animal Care and Use Committee (IACUC) of China Medical University and the care of animal was carried out according to the Guiding Principles for the Care and Use of Laboratory Animals. They were housed for at least 1 week before starting experiment in a temperature-(23 ± 1°C) and humidity-(60%) regulated environment with free access to standard food in pellets and tap water, on a 12 h - 12 h light/dark cycle (light phase: 08:00 to 20:00 h) was maintained. After 1 week of acclimatization, eight rodents each group in the below experiments were used. Then, the below drugs were administered, and the analgesic and anti-inflammatory assays were operated by double-blind method. After behavioral measurement, all animals were killed by carbon dioxide.

### Measurement of tetrandrine in FJHQT by HPLC system

The determination of tetrandrine from FJHQT extract was carried out by HPLC with a photodiode array detector. The HPLC system was consisted of a Shimadzu LC-20AT solvent delivery system, equipped with a SPD-M20A photodiode array detector, set at 263 nm. Samples were injected with SiL-20A autosampler to separate on the TSK-Gel ODS-100S column. All chromatographic operations were carried out at 25°C. The mobile phase consisted of solvent A (0.3% formic acid) and solvent B (acetonitrile). The elution profile for A was 0–10 min, linear gradient change of 0 - 5%; 10–40 min, linear gradient change to 55%; and maintained for another 10 min with a post run time in order to equilibrate the column and for the baseline to return to the normal and initial working conditions. Flow rate was 1.0 mL/min. FJHQT extract was dissolved in methanol and then filtered with a 0.22 μm filter. Stock solutions of the standards were prepared in methanol to final concentrations of 1 mg/mL. Ten μL of standard or sample solutions was injected into the HPLC instrument for analysis in triplicate. The chromatographic peak of tetrandrine was confirmed by comparing their retention times and UV spectra.

### Acetic acid-induced abdominal writhing response in mice

Each mouse was given intraperitoneally 1% aqueous solution of acetic acid (10 mL/kg body weight), and then was placed in the individual observation boxes. Five minutes after the injection of acetic acid, the number of writhing responses per mouse was counted for 10 minutes during acetic acid-induced abdominal writhing [7]. Finally, the number of writhing responses permitted us to express the percentage of protection using the following ratio: (Control mean - treated mean) × 100 / control mean.

### Formalin-induced licking response in mice

This method represented a modification of that described by Shibata et al. [8]. Each mouse was placed in the observation chamber on an acrylic transparent plate floor for 5 min prior to the formalin injection. Beneath the floor, a large mirror was inclined at a 45° angle in order to allow clear observation of the paws of the animal. The animals were administered 25 μL of 1% formalin into the right subplantar. Then, each animal was returned to the chamber and the two distinct periods of the intensive licking response was observed. The first period (early phase) was recorded 0–5 min after formalin injection and the second period (late phase) was recorded 10–35 min after formalin. The time (in seconds) spent in licking responses of the injected paw was measured as an indicator of pain response.

### Carrageenan-induced paw edema in rats

The anti-inflammatory activity was determined in rats by measuring the mean increase in hind paw volume after the subplantar injection of carrageenan [9]. The animals were injected with 0.1 mL 1% carrageenan in the right hind foot under the plantar aponeurosis. The inflammation was quantitated in terms of milliliters using a plethysmometer (7150 Ugo Basile) which recorded small differences in water level caused by volume displacement. Before any treatment, the average volume of the backpaws of each animal was determined (V_o_), after 3 measurements which did not differ from more than 4% (preciseness of the apparatus). Then 30, 60, 90, 120, 150, 180, 210, 240 min after carrageenan injection, the average volume of the backpaws of each animal was determined (V_t_), after 3 measurements which did not differ from more than 4%. The percentages of edema at each record were calculated by compared the average volume of the backpaws of each animal (V_t_) after carrageenan injection with the average volume of the backpaws of each animal (V_o_) before any treatment. Percentages of inhibition were obtained for each group by using the following ratio: [(V_t_ - V_o_)control - (V_t_ - V_o_)treated] × 100 / (V_t_ - V_o_)control.

### Subacute toxicity study in rats

The 28-day repeated oral toxicity studies were carried out in rats according to the OECD test guideline 407 [10]. Rats were divided randomly into 4 groups of 8 animals each. After an overnight fast, control group which rats received sterile distilled water, whereas other groups which rats received FJHQT extract at the doses of 0.1, 0.5, and 1.0 g/kg body weight, respectively. Doses of FJHQT extract were administered daily by oral gavage in the volume of 10 mL/kg body weight, once daily for 28 consecutive days. The rats were observed daily for any abnormal clinical signs and death during the study period. Body weight and food intake were measured and recorded daily during the study period. At the end of the study, all animals fasted overnight and, on 29th day, the animals were weighed. Blood was collected from retroorbital technique with or without EDTA for hematological and biochemical analysis, respectively. The animals were sacrificed and other body organs were taken out for detailed weight and histopathological changes.

### Hematological parameters and biochemical estimations

Red blood cells (RBC), white blood cells (WBC), hematocrit (HCT), hemoglobin (HGB), mean corpuscular hemoglobin (MCH), mean corpuscular hemoglobin concentration (MCHC), mean corpuscular volume (MCV), and platelet counts [11] were determined in control and FJHQT extract-treated groups. The serum was carefully aspirated into sample bottles for the various biochemical assays. Assay kits for aspartate transaminase (AST), alanine transaminase (ALT), creatinine, blood glucose, blood urea nitrogen (BUN), total protein and albumin analysis were purchased from Radox diagnostic kit and determined in the serum following the procedure described in the kits.

### Organs weight and histology

The rats were quickly dissected and the brain, heart, lung, liver, spleen, kidney, adrenal, and testis were excised and weighed. The specimens for histopathology were fixed in 10% neutral, buffered formalin for 18 h at 4°C. Thickness (3–4 μm) of each specimen of liver and kidney was cut and stained with hematoxylin and eosin stain following the standard laboratory procedures. The stained sections were examined under microscope for any cellular damage or change in morphology of that particular tissue.

### Statistical analysis

All data obtained during the antinociceptive and anti-inflammatory activity was expressed in terms of mean and standard errors, and further analyzed by using ANOVA one-way analysis of variance, followed by Scheff’s test. When probability (*P*) was less than 0.05, the difference was considered to be significant.

## Results and discussion

### Identification of Radix Stephania Tetrandra and measurement of tetrandrine content in FJHQT extract

**Figure 1:**
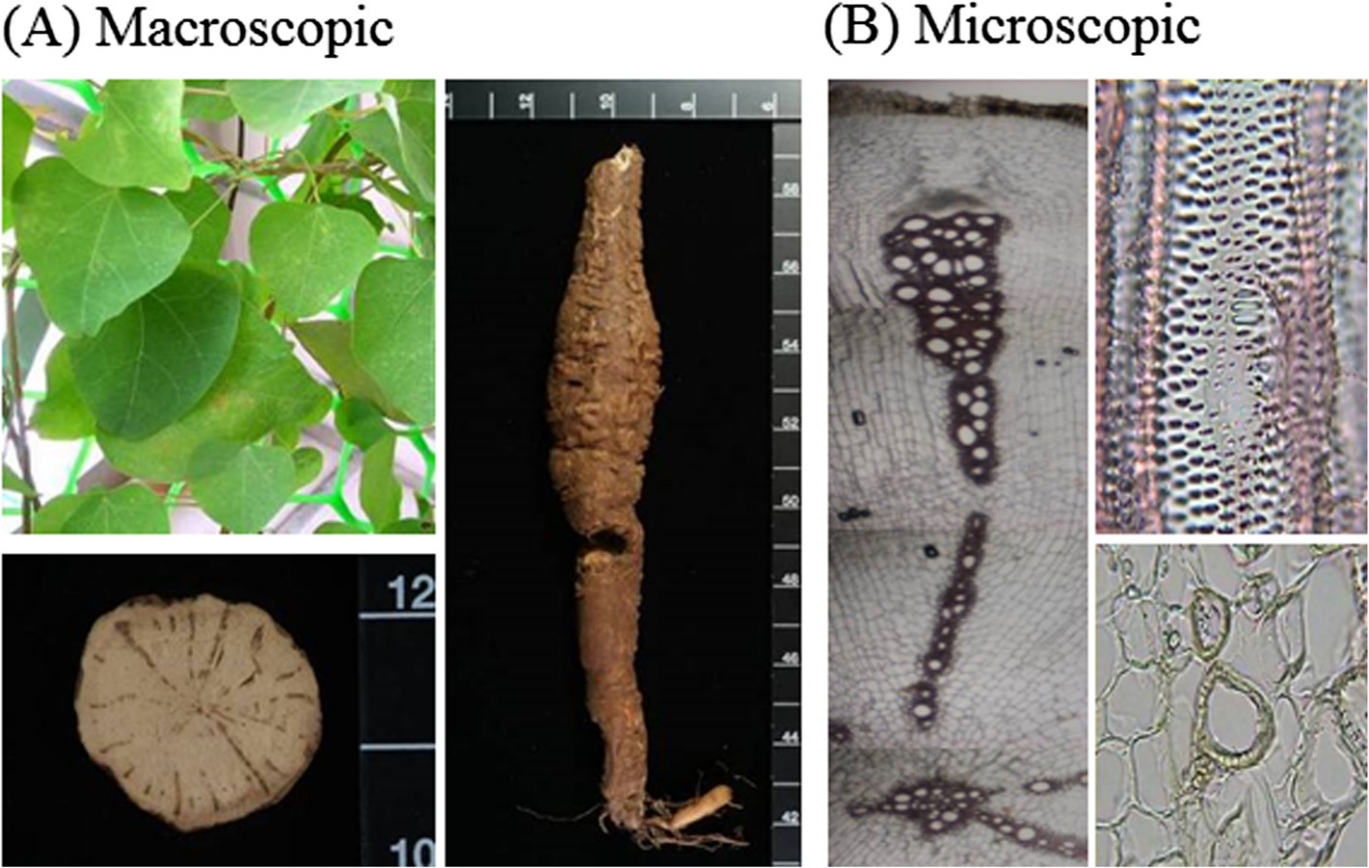
**Pharmacognostic photographs of Radix Tetrandria. (A)** Macroscopic characteristics **(B)** Microscopic characteristics.

**Figure 2:**
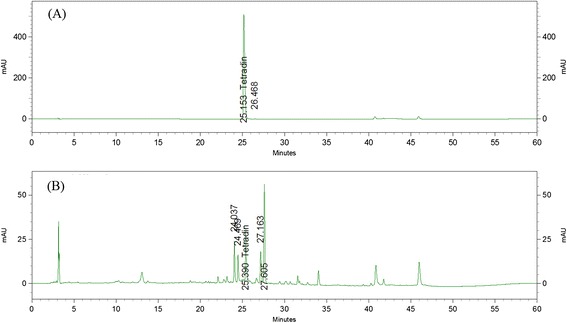
**HPLC chromatograms of aqueous extract of Fang-Ji-Huang-Qi-Tang at 263 nm. (A)** Standard, **(B)** Fang-Ji-Huang-Qi-Tang.

Due to the misuse between Radix Stephania Tetrandra and Aristolochia species, we first identified Radix Stephania Tetrandra before the preparation of FJHQT extract with macroscopic and microscopic observation in medicinal material of Radix Stephania Tetrandra. The photographs of macroscopic characteristics including original plant and medicinal material of Radix Stephania Tetrandra were shown in Figure 1 (A). The oval or irregular stone cells in cortex and pits in round or oval reticulate vessels were found in the microscopic observation of Radix Stephania Tetrandra (Figure 1 (B)). These observed characteristics were same as the photographs of Radix Stephania Tetrandra in the literature [12]. The photographs of macroscopic and microscopic characteristics of other medicinal materials were shown in Additional file 1. We further quantified the content of tetrandrine in FJHQT extract after the preparation of FJHQT extract with HPLC. The chromatographs of tetrandrine and FJHQT extract were shown in Figure 2. Retention time of tetrandrine in HPLC chromatogram at 263 nm is about at 25.15 min. The content of tetrandrine in FJHQT extract is about 2.5 ± 0.2 mg/g dry weight in accordance with the calibration curve of tetrandrine.

### Antinociceptive and anti-inflammatory activities of FJHQT extract

**Figure 3:**
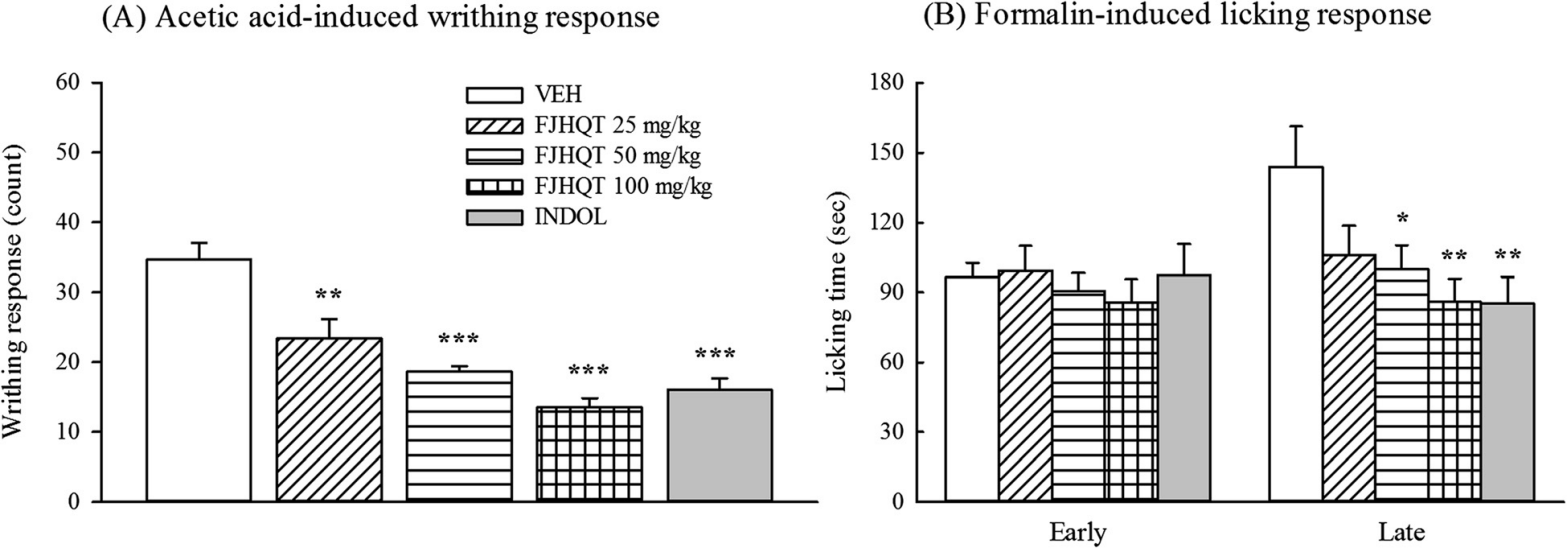
**Effect of Fang-Ji-Huang-Qi-Tang extract (FJQHT, 25, 50 and 100 mg/kg) and indomethacin (INDO, 10 mg/kg) on (A) the acetic acid-induced writhing response, and (B) the early (0–5 min) and late phase (10–35 min) of formalin-induced licking response in mice.** Each values are represented as mean ± S.E. (N = 8). *P < 0.05. ** P < 0.01. *** P < 0.001 as compared with the VEH group.

Acetic acid-induced abdominal writhing response and formalin-induced licking response are very useful models for assessing the effects of antinociceptive drugs. To evaluate the antinociceptive activities of FJHQT extract, we performed the acetic acid-induced abdominal writhing response and formalin-induced licking response in mice. Acetic acid-induced abdominal writhing response is mainly based on the peripheral system, which involves prostaglandin synthesis via cyclooxygenase [13]. Our present result found pretreatment with FJHQT extract (25–100 mg/kg, po) decreased acetic acid-induced writhing response in a dose-dependent manner in mice (Figure 3(A)) (*P* < 0.01, *P* < 0.001). Indomethacin, a positive control, at 10 mg/kg also decreased acetic acid-induced writhing response (Figure 3(A)) (*P* < 0.001). Thus, FJHQT extract possessed antinociceptive effect against the acetic acid-induced abdominal writhing response in mice. We further found that FJHQT extract (25–100 mg/kg, po) also significantly prevented the late but not early phase of formalin-induced licking response in mice (Figure 3(B)) (*P* < 0.05, *P* < 0.01). Indomethacin also effectively inhibited the late but not early phase of formalin-induced licking response in mice (Figure 3(B)) (*P* < 0.01). In fact, some components of FJHQT extract such as Radix Stephania Tetrandra and Rhizoma Zingiberis have been proven to possess the antinociceptive activities in mice [14,15]. Moreover, recent report indicated that tetrandrine, an active ingredient of Radix Stephania Tetrandra, possessed the antinociceptive effect in mice [16]. Therefore, this result, confirmed the clinical use for the painful symptoms, showed that FJHQT extract produced pronounced and dose-related antinociceptive activities against acetic acid-induced writhing responses and the late phase of formalin-induced licking responses. Furthermore, there are obvious differential properties in the early and late phase of formalin-induced licking responses that the early phase is caused by central nerve fiber activation and the late phase is dependent on the functional changes in the peripheral nerves [8]. Thus, we suggested that the antinociceptive mechanism of FJHQT extract might be through the peripheral systems of pain pathway, in consistence with indomethacin.

**Figure 4:**
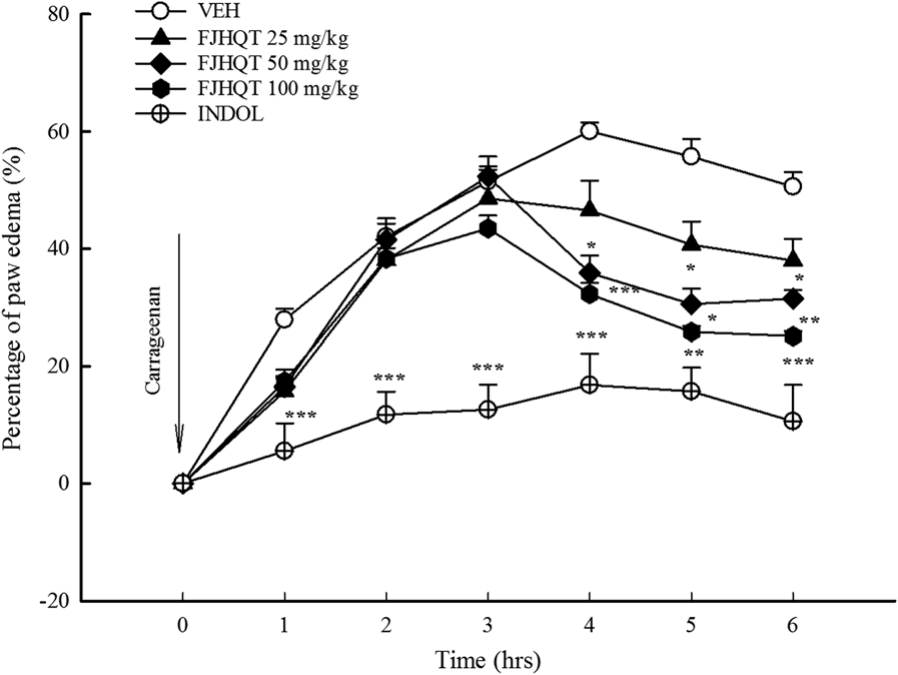
**Effect of Fang-Ji-Huang-Qi-Tang extract (FJQHT, 25, 50 and 100 mg/kg) and indomethacin (INDO, 10 mg/kg) on the carrageenan-induced paw edema in rats.** Each values are represented as mean ± S.E. (N = 8). * *P* < 0.05. ** *P* < 0.01,as compared with the VEH group.

Due to FJHQT extract only inhibited the late phase of formalin-induced licking response and early reports indicated the late phase seems to be an inflammatory response with inflammatory pain [8], we further investigated the anti-inflammatory effect of FJHQT extract with carrageenan-induced paw edema in rats. We found that pretreatment with FJHQT extract at 50–100 mg/kg inhibited carrageenan-induced edema formation from 4 to 6 hours after carrageenan injection in rats (Figure 4) (*P* < 0.05, *P* < 0.01, *P* < 0.001). Indomethacin at 10 mg/kg, a positive control, also effectively inhibited carrageenan-induced edema formation throughout the measurement period in rats (Figure 4) (*P* < 0.001). Previous researchers have indicated that almost all components of FJHQT extract possessed anti-inflammatory activities *in vitro* and *in vivo* [17-22]. Tetrandrine also possessed anti-inflammatory activities *in vitro* and *in vivo* [23-26]. Thus, this result, also confirmed the clinical use for the inflammatory disorders such as rheumatoid arthritis, showed that FJHQT extract also produced pronounced and dose-related anti-inflammatory activity against carrageenan-induced paw edema. All components of FJHQT extract produced a synergistic effect on the anti-inflammatory activity of FJHQT extract. Tetrandrine is also a major active ingredient of Radix Stephania Tetrandra and FJHQT extract.

Shibata et al. [8] suggested the early phase of formalin-induced licking response was related to bradykinin and substance P, and the late phase was related to bradykinin, autocirnes and prostaglandin. Moreover, previous researchers have indicated that the mechanism of carrageenan-induced edema usually separates into three phases. First phase (1.5 h after carrageenan treatment) is related to autocrines and PAF. Second phase (from 1.5 h to 2.5 h after carrageenan treatment) is related to kinins. Third phase (2.5 h after carrageenan treatment) is related to prostaglandins and leukotrienes [27-29]. From our above results in three models, the antinociceptive and anti-inflammatory mechanism of FJHQT extract might be mainly related to bradykinin, autocirnes or prostaglandin pathway because FJHQT extract mainly inhibited the late-phase of formalin-induced licking response and the third phase of carrageenan-induced paw edema. Refereeing to the reports of all components of FJHQT extracts, their anti-inflammatory mechanism might be mainly related to NF-kappaB, iNOS, cyclooxygenase-2 (COX-2) / prostaglandin (PGE) pathway [2,19,30,31]. Furthermore, some researchers indicated that tetrandrine also possessed the anti-inflammatory effect *via* the inhibition of NF-kappa / COX-2 pathway, the release of pro-inflammatory cytokines including IL-1β, IL-6 and TNF-α in mice [16,24,25,32]. Therefore, our present results, in consistence with these literatures of all medicinal components and tetrandrine, demonstrated that the anti-inflammatory effects of FJHQT extract might be mainly through NF-kappaB, iNOS, cyclooxygenase-2 (COX-2)/prostaglandin (PGE) pathway, and then modulating the release of pro-inflammatory cytokines.

### Sub-acute toxicology of FJHQT extract

**Figure 5:**
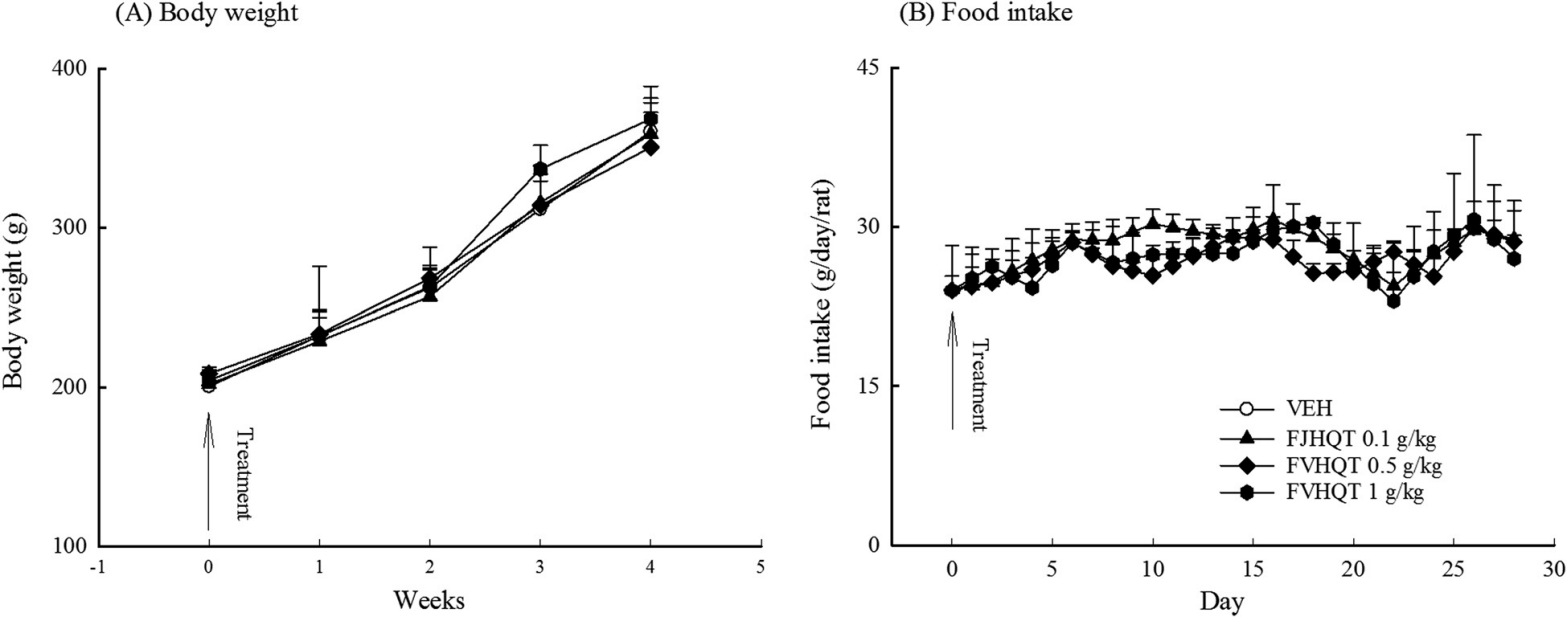
**Effect of Fang-Ji-Huang-Qi-Tang extract (FJQHT, 0.1, 0.5 and 1.0 g/kg) on (A) the tendency of body weight and (B) daily food intake during 28-day repeated treatment in rats.** Each value are represented as mean ± S.E. (N = 8).

**Figure 6:**
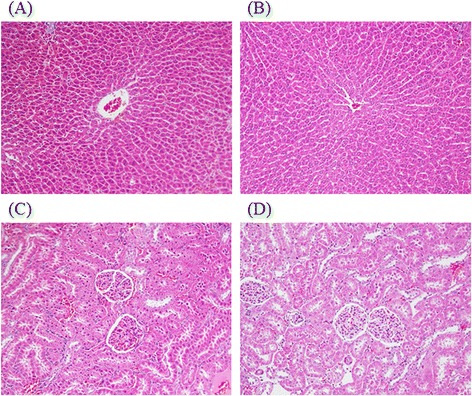
**Histology of liver and kidney (H&E stain, 100x) in rats. (A)** and **(C)** Section of liver and kidney from vehicle-treated rats; **(B)** and **(D)** Section of liver and kidney from Fang-Ji-Huang-Qi-Tang (FJQHT, 1.0 g/kg)-treated rats.

Due to the misuse between Radix Stephania Tetrandra and Aristolochia species, Aristolochia species often cause acute or chronic nephropathy via aristolochic acid. Thus, we evaluated the subacute toxicology of FJHQT after 28-day oral administration in rats although we have identified the corrected use of Radix Stephania Tetrandra in FJHQT extract. Rats treated with FJHQT extract at 0.1, 0.5, or 1.0 g/kg body weight daily for 28 day, were survival and normal throughout the administration. FJHQT extract-treated rats did not show any changes in general behavior or other physiological activities. No change was observed in body weight and food intake in FJHQT extract (0.1, 0.5, or 1.0 g/kg)-treated groups compared with control group after 28 days-repeated treatment in rats (Figure 5(A) and (B)). Secondly, there were not significantly different between FJHQT extract (0.1, 0.5, or 1.0 g/kg)-treated rats and control rats in all hematological parameters including RBC, HGB, HCT, MCV, MCH, MCHC, WBC, and platelet counts (Table 1). Biochemical parameters for liver and kidney function test such as AST, ALT, creatinine, BUN, blood glucose, total protein, and albumin of FJHQT extract (0.1, 0.5, or 1.0 g/kg)-treated rats did not show any difference with those of control group (Table 2). The urine parameters including volume, pH value, protein and glucose of FJHQT extract (0.1, 0.5, or 1.0 g/kg)-treated rats did not show any difference with those of control group (Table 3). Finally, there were no difference between FJHQT extract (0.1, 0.5, or 1.0 g/kg)-treated rats and control rats in their mean weights and gross examinations of major organs including brain, heart, lung, liver, spleen, kidney, adrenal, and testis dissected from all rats (Table 4). When administered with higher dose of 1.0 g dose of FJHQT extract for 28 days, no histopathological changes in liver and kidney were observed (Figure 6). Thus, we suggested that FJHQT extract at 10 time effective dose for 28-day repeated oral administration did not cause any toxicological responses and histopathological changes if Radix Stephania Tetrandra in the component of FJHQT was not misused with Aristolochia species. Moreover, other report indicated tetrandrine at 50 mg/kg for 3-month repeated intraperitoneal administration caused death and moderate hydropic degeneration of the distal tubules in the kidneys in mice [33]. Thus, we elaborated that FJHQT extract has higher safety index in its antinociceptive and anti-inflammatory activities because the content of tetrandrine in the used dosage of FJHQT extract for the antinociceptive and anti-inflammatory tests is about 25 – 250 μg/g body weight (thousandth of chronic toxicological dose).

**Table 1:** **Effects of Fang-Ji-Huang-Qi-Tang (FJQHT, 0.1, 0.5, 1.0 g/kg) after 28-day repeated oral administration on hematological parameters in rats**

**Groups**	**RBC (10** ^**6**^ **)**	**HGB (g/dL)**	**MCV (fj)**	**MCH (pg)**	**MCHC (g/dL)**	**HCT (%)**	**WBC (10** ^**3**^ **)**	**Platelet (10** ^**3**^ **)**
Normal	7.5 ± 0.2	14.5 ± 0.2	61.7 ± 0.4	19.4 ± 0.1	31.6 ± 0.1	45.9 ± 0.7	7.7 ± 0.7	1076.1 ± 55.6
FJHQT 0.1 g/kg	7.3 ± 0.2	15.0 ± 0.4	62.7 ± 1.3	20.1 ± 0.3	31.7 ± 0.3	44.8 ± 0.8	7.7 ± 0.5	1141.1 ± 50.2
FJHQT 0.5 g/kg	7.3 ± 0.1	14.5 ± 0.2	61.6 ± 0.9	20.0 ± 0.4	31.8 ± 0.2	47.9 ± 1.2	7.7 ± 0.5	1112.9 ± 57.7
FJHQT 1.0 g/kg	7.7 ± 0.1	14.8 ± 0.2	61.2 ± 0.6	19.3 ± 0.2	31.6 ± 0.2	46.9 ± 0.7	7.7 ± 0.7	1184.4 ± 66.9

Data are represented with mean ± SEM, N = 8.

**Table 2:** **Effects of Fang-Ji-Huang-Qi-Tang (FJQHT, 0.1, 0.5, 1.0 g/kg) after 28-day repeated oral administration on plasma biochemical parameters in rats**

**Groups**	**Glu (mg/dL)**	**TP (mg/dL)**	**Albumin (mg/dL)**	**Globulin (mg/dL)**	**ALT (U/L)**	**AST (U/L)**	**BUN (mg/dL)**	**Creatinine (mg/dL)**
Normal	80.6 ± 8.8	7.2 ± 0.9	4.2 ± 0.6	3.0 ± 0.3	34.0 ± 4.9	12.9 ± 1.8	14.0 ± 1.2	0.8 ± 0.1
FJHQT 0.1 g/kg	85.4 ± 8.3	7.7 ± 0.9	4.5 ± 0.6	3.2 ± 0.3	32.1 ± 3.2	13.4 ± 1.4	13.5 ± 1.7	0.9 ± 0.1
FJHQT 0.5 g/kg	81.0 ± 4.6	7.5 ± 0.3	4.6 ± 0.1	2.9 ± 0.2	39.0 ± 2.9	14.8 ± 1.1	14.2 ± 0.8	0.7 ± 0.2
FJHQT 1.0 g/kg	82.4 ± 3.0	7.3 ± 0.3	4.4 ± 0.2	2.9 ± 0.1	38.1 ± 1.0	15.1 ± 0.9	13.8 ± 1.0	0.7 ± 0.1

Data are represented with mean ± SEM, N = 8.

**Table 3:** **Effects of Fang-Ji-Huang-Qi-Tang (FJQHT, 0.1, 0.5, 1.0 g/kg) after 28-day repeated oral administration on urine parameters in rats**

**Groups**	**Volumes (ml)**	**pH value**	**Protein (mg/L)**	**Glucose (mmol/L)**
Normal	22.0 ± 2.5	6.8 ± 0.1	35.3 ± 2.7	6.8 ± 1.5
FJHQT 0.1 g/kg	21.4 ± 2.5	7.1 ± 0.1	39.9 ± 3.5	7.0 ± 0.8
FJHQT 0.5 g/kg	19.1 ± 3.3	7.0 ± 0.3	41.4 ± 5.4	6.9 ± 1.0
FJHQT 1.0 g/kg	21.0 ± 2.4	7.1 ± 0.1	40.8 ± 5.9	6.7 ± 0.7

Data are represented with mean ± SEM, N = 8.

**Table 4:** **Effects of Fang-Ji-Huang-Qi-Tang (FJQHT, 0.1, 0.5, 1.0 g/kg) after 28-day repeated oral administration on organ weight in rats**

**Groups**	**Brain (g)**	**Heart (g)**	**Lung (g)**	**Liver (g)**	**Spleen (g)**	**Kidney (g)**	**Adrenal (mg)**	**Testis (g)**
Normal	1.96 ± 0.03	1.25 ± 0.05	1.40 ± 0.07	9.37 ± 0.44	0.70 ± 0.04	2.50 ± 0.08	53 ± 4	2.91 ± 0.03
FJHQT 0.1 g/kg	1.96 ± 0.03	1.30 ± 0.04	1.65 ± 0.14	9.24 ± 0.24	0.71 ± 0.05	2.52 ± 0.06	53 ± 4	3.08 ± 0.16
FJHQT 0.5 g/kg	1.97 ± 0.02	1.21 ± 0.04	1.38 ± 0.07	9.02 ± 0.45	0.64 ± 0.04	2.43 ± 0.11	52 ± 4	2.91 ± 0.03
FJHQT 1.0 g/kg	2.03 ± 0.05	1.32 ± 0.04	1.54 ± 0.14	9.45 ± 0.22	0.65 ± 0.04	2.55 ± 0.08	45 ± 5	3.18 ± 0.12

Data are represented with mean ± SEM, N = 8.

## Conclusion

From our present results, FJHQT extract is a very safe and pronounced antinociceptive and anti-inflammatory Chinese prescription. These pharmacological activities of FJHQT extract confirms the clinical use for the painful symptoms caused by inflammatory disorders such as rheumatoid arthritis. The antinociceptive and anti-inflammatory activities of FJHQT extract were from the synergic effects of its medicinal components because many researchers have evidenced all medicinal components possessed antinociceptive and anti-inflammatory activities *in vitro* and *in vivo* [14,15,17-22], especially Radix Stephania Tetrandra. Furthermore, we reviewed the antinociceptive and anti-inflammatory literatures of these medicinal components of FJHQT extract and found that their antinociceptive and anti-inflammatory mechanisms were due to the inhibition of NF-kappa B / COX-2 / iNOS pathway and the decrease of pro-inflammatory cytokines secretion such as IL-1β and TNF-α [2,19,30,31]. Even the antinociceptive and anti-inflammatory activities of tetrandrine also were through the inhibition of NF-kappa / COX-2 pathway, the release of pro-inflammatory cytokines including IL-1β, IL-6 and TNF-α in mice [16,24,25,32]. Hence, we speculated the antinociceptive and anti-inflammatory mechanisms of FJHQT extract might be same as those of its medicinal components which be related to NF-kappa B / COX-2 / iNOS pathway and the release of pro-inflammatory cytokines, but this speculation should be investigated in the future.

## Additional file

Additional file 1: Figure S1. Pharmacognostic photographs of Radix Astragali. (A) Macroscopic characteristics (B) Microscopic characteristics. **Figure S2.** Pharmacognostic photographs of Rhizoma Atractylodis Macrocephalae. (A) Macroscopic characteristics (B) Microscopic characteristics. **Figure S3.** Pharmacognostic photographs of Radix Glycyrrhizae. (A) Macroscopic characteristics (B) Microscopic characteristics. **Figure S4.** Pharmacognostic photographs of Rhizoma Zingiberis. (A) Macroscopic characteristics (B) Microscopic characteristics. **Figure S5.** Pharmacognostic photographs of Fructus Ziziphi Jujubae. (A) Macroscopic characteristics (B) Microscopic characteristics.

## Abbreviations

ALT: Alanine transaminase
AST: Aspartate transaminase
BUN: Blood urea nitrogen
FJHQT: Fang-Ji-Huang-Qi-Tang
HCT: Hematocrit
HGB: Hemoglobin
HPLC: High performance liquid chromatography
INDO: Indomethacin
MCH: Mean corpuscular hemoglobin
MCHC: Mean corpuscular hemoglobin concentration
MCV: Mean corpuscular volume
RBC: Red blood cells
WBC: White blood cells
