# Supplementary material for: Anti-nociceptive, anti-inflammatory and toxicological evaluation of Fang-Ji-Huang-Qi-Tang in rodents
Source: BMC Complement Altern Med. 2015 Feb 5;15:10. doi: 10.1186/s12906-015-0527-5 (PMC4322796; doi:10.1186/s12906-015-0527-5)
Supplement: Additional file 1: Figure S1. — Pharmacognostic photographs of Radix Astragali. (A) Macroscopic characteristics (B) Microscopic characteristics. Figure S2. Pharmacognostic photographs of Rhizoma Atractylodis Macrocephalae. (A) Macroscopic characteristics (B) Microscopic characteristics. Figure S3. Pharmacognostic photographs of Radix Glycyrrhizae. (A) Macroscopic characteristics (B) Microscopic characteristics. Figure S4. Pharmacognostic photographs of Rhizoma Zingiberis. (A) Macroscopic characteristics (B) Microscopic characteristics. Figure S5. Pharmacognostic photographs of Fructus Ziziphi Jujubae. (A) Macroscopic characteristics (B) Microscopic characteristics. [file 12906_2015_527_MOESM1_ESM.docx]

(A) Macroscopic

(B) Microscopic


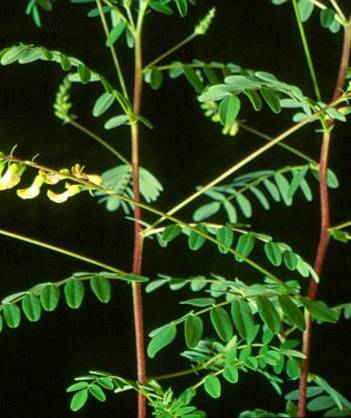

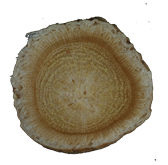

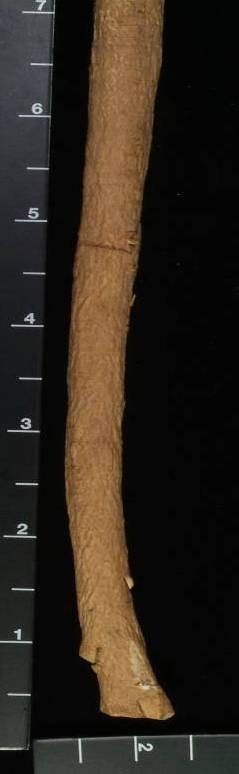

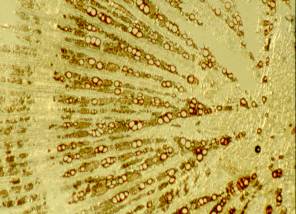

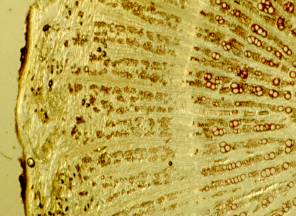

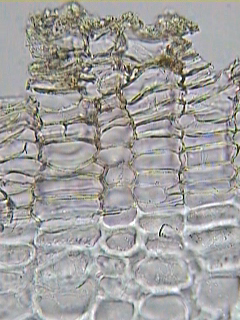

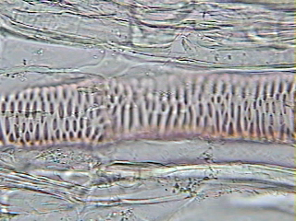


**Figure S1. Pharmacognostic photographs of Radix Astragali.** (A) Macroscopic characteristics (B) Microscopic characteristics.

(A) Macroscopic

(B) Microscopic


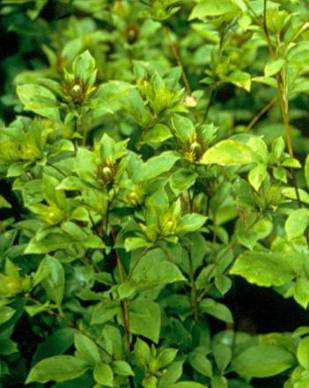

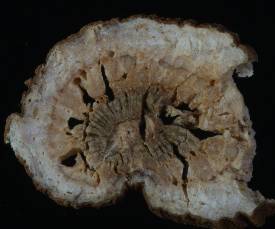

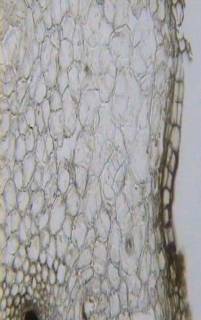

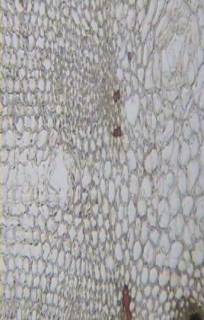

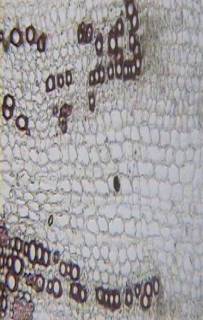

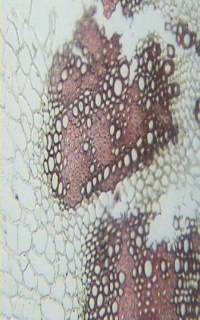

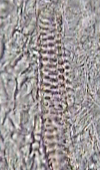

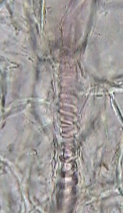

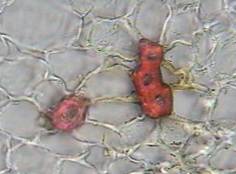


**Figure S2. Pharmacognostic photographs of Rhizoma Atractylodis Macrocephalae.** (A) Macroscopic characteristics (B) Microscopic characteristics.

(A) Macroscopic

(B) Microscopic


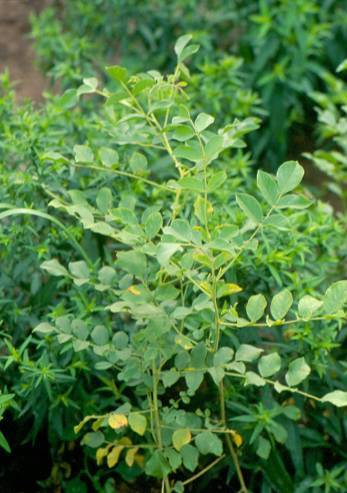

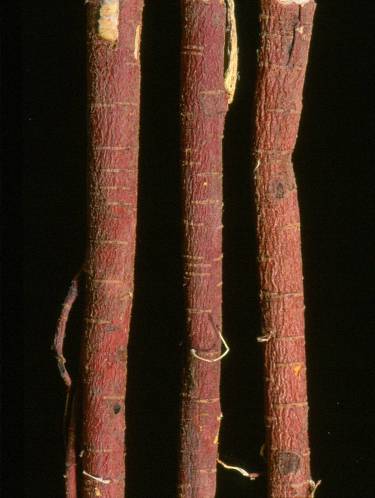

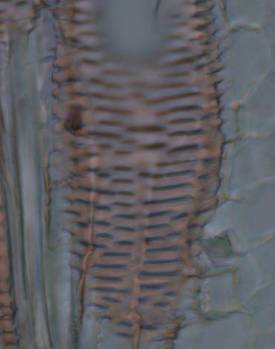

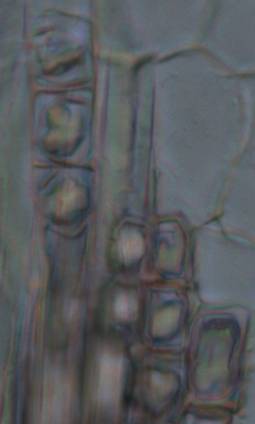

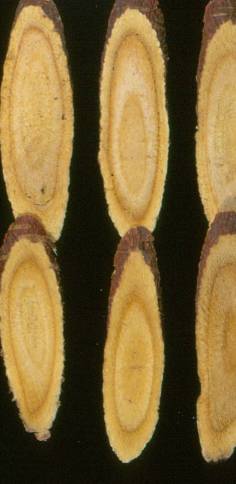


**Figure S3. Pharmacognostic photographs of Radix Glycyrrhizae.** (A) Macroscopic characteristics (B) Microscopic characteristics.

(A) Macroscopic

(B) Microscopic


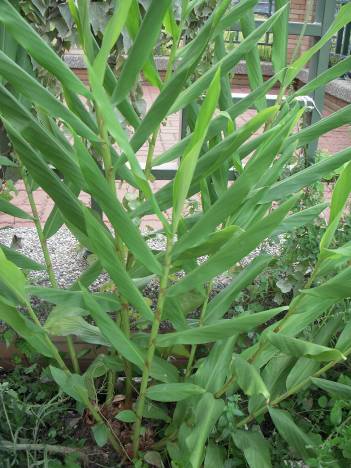

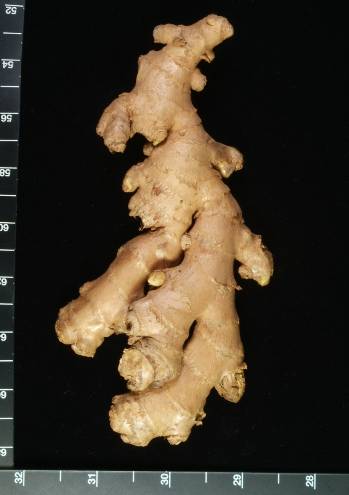

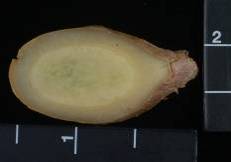

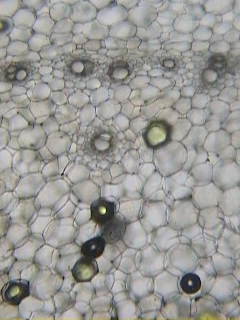

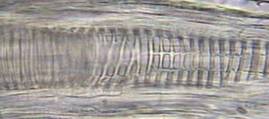

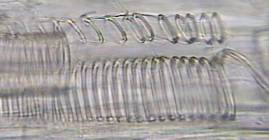

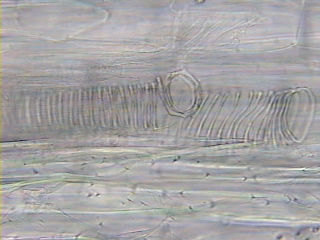


**Figure S4. Pharmacognostic photographs of Rhizoma Zingiberis.** (A) Macroscopic characteristics (B) Microscopic characteristics.

(A) Macroscopic

(B) Microscopic


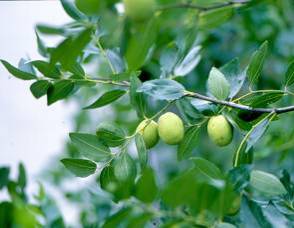

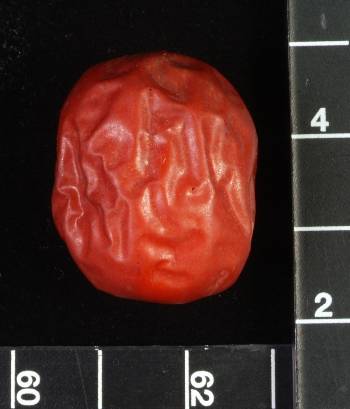

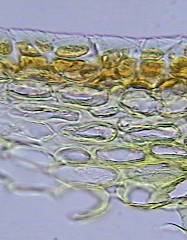

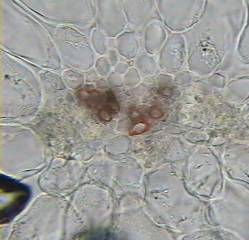


**Figure S5. Pharmacognostic photographs of Fructus Ziziphi Jujubae.** (A) Macroscopic characteristics (B) Microscopic characteristics.
